# Zinc Oxide-Doped Antibacterial Soda Lime Glass Produced as a Glass Container

**DOI:** 10.1021/acsomega.2c07469

**Published:** 2023-03-03

**Authors:** Barış Demirel, Melek Erol Taygun

**Affiliations:** † Department of Chemical Engineering, Istanbul Technical University, Maslak, 34469 Istanbul, Turkey; ‡ Sisecam Science Technology and Design Center, Gebze 41400, Kocaeli, Turkey

## Abstract

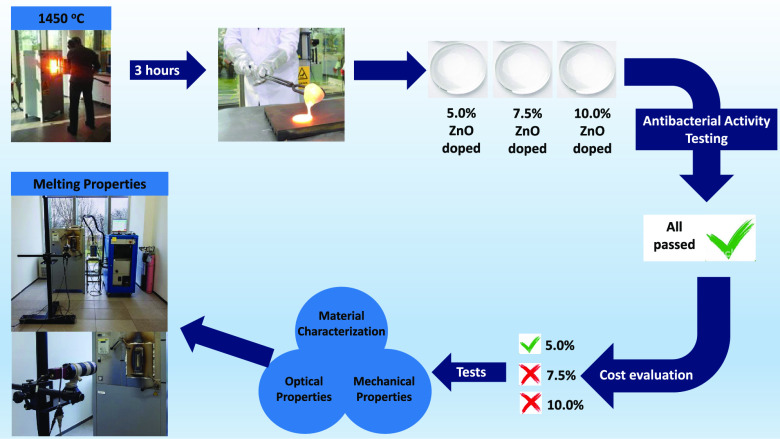

The aim of this study is to produce and characterize glass materials, which have an enhanced antibacterial property by the conventional melting method. Container glass compositions including different amounts of zinc oxide (ZnO) (5.0, 7.5, and 10.0%) were prepared and melted to be able to obtain the antibacterial glass. The Release and antibacterial tests, which were performed after the melting process, showed that the glass doped with 5% ZnO was the most appropriate composition according to test results (99.82% *Escherichia coli* inactivation) and its raw materials’ costs. Physical, thermal, and mechanical properties such as thermal expansion coefficient (86.1 × 10^–7^/°C), density (2.523 g/cm^3^), refractive index (1.5191), hardness (596 kg/mm^2^), and elastic modulus (5.84 GPa) of the glass doped with 5% ZnO were determined, and the results showed that the obtained antibacterial glass sample is suitable to be used as a glass container. HighTemperature Melting Observation System studies were performed on the produced antibacterial glass composition, and it was found that the antibacterial glass can be produced in soda lime glass furnaces without changing any furnace design and production parameters. This antibacterial glass can be a remarkable product for the pharmaceutical and food industries.

## 1 Introduction

Health has always been the most important research area in science and technology for humanity. In this studies, it is aimed to reduce or completely destroy bacteria, which is one of the main causes of all infections and diseases. The most effective solution to these bacteria that threaten human health is creating antibacterial agents. For this purpose, antibacterial properties have been gained in ceramic^1,2^ and polymeric materials^3,4^ that people use frequently in their daily lives. Furthermore, adding an antibacterial effect to the glass materials^5−7^ that are frequently used for buildings,^8^ cars, tableware, food, and medicine^9^ has become important and scientific studies have been increasing day by day in this regard.

In the literature, antibacterial glass can be obtained by using the ion-exchange process,^10^ sol–gel technique,^11^ or glass surface coating.^12^ Antibacterial glass production processes require additional process equipment and changing the process parameters, which cost a lot. Another big problem with those processes are the peeling problem and not long lasting antibacterial behavior. An alternative method is adding metal ions with an antibacterial effect to the batch. Silver is the most used ion for this method for glass microsphere,^13^ bioactive glass,^14^ or soda lime glass.^15^

**Figure 1:**
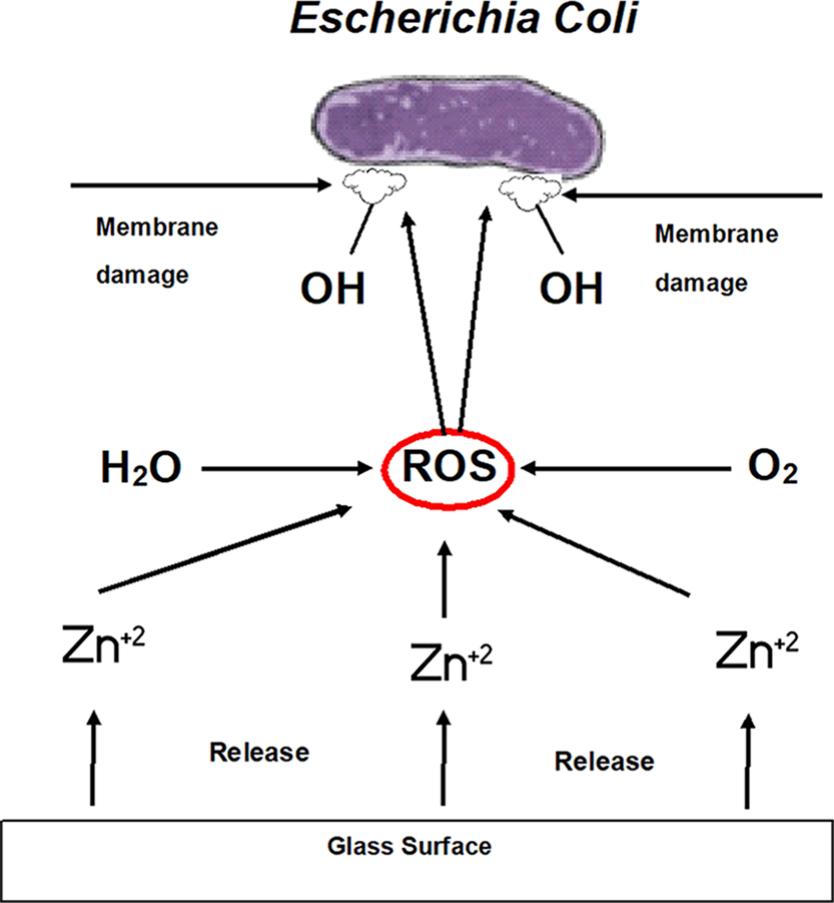
Antibacterial mechanism of zinc oxide from the glass surface.

The antibacterial effect of zinc oxide is differentiated from biocide agents (like silver) in that it has no environmental effects^16,17^ and toxicity.^18−21^ In the literature, there are studies where zinc oxide (ZnO) was added to tellurite^22^ and borate glass^23^ systems. Also, ZnO has been used to gain antibacterial and photocatalytic feature to glass ceramic systems or different type material systems.^24−26^ ZnO stimulates oxidative stress via generation of reactive oxygen species (ROS) in bacterial cells, which arrests cell growth and brings about cell lysis with membrane damage.^27^ A schematic representation of the antibacterial mechanism to *Escherichia coli* of glass samples doped with the ZnO sample is shown in Figure 1. However, very few studies related to the incorporation of zinc into a glass batch were performed by using the classical melting process in the literature.^28−31^

Soda lime glasses have many areas of use, from the window we touch to the packaging of the water we drink. Glass packaging, which has a critical place in human life, has a market share of 46%.^32^ Adding antibacterial properties to these materials, which have such a wide area of use and are in close contact with food and drinks, is beneficial for human health. Although there have been many studies on antibacterial properties of bioactive glasses, the data on antibacterial properties of soda lime glasses is very limited, covering soda lime glasses produced with the ion exchange process. In this study, it was aimed to produce first antibacterial soda lime glass doped with ZnO by using the classical melting method suitable in the glass market (by application hospitals, food and beverage, household/residential). This procedure enables to produce soda lime glass with antibacterial properties, without any additional processes. It means that no extra operational and investment costs will be needed. Furthermore, the short term antibacterial effect and the peeling problems encountered in the antibacterial glass products obtained by ion exchange, sol–gel, and coating methods can be eliminated with antibacterial soda lime glass doped with ZnO by using the classical melting method. The structural, mechanical, optical, and physical properties of the obtained antibacterial glass were investigated in detail to be able to determine its application area. Furthermore, the High Temperature Melting Observation System (HTMOS) was also used for the first time on the obtained antibacterial glass sample to determine melting behavior of it.

## 2 Experimental Study

### 2.1 Glass Composition and Melting

In food and pharmaceutical industries, antibacterial properties are becoming more important. Therefore, zinc oxide (ZnO) was selected as a doping agent having no toxic properties in the glass batch. A typical glass container composition used in this study is shown in Table 1.

**Table 1:** Typical Glass Container (Non-Antibacterial) Composition (in wt. %)

SiO_2_ (%)	Al_2_O_3_ (%)	Fe_2_O_3_ (%)	TiO_2_ (%)	CaO (%)	MgO (%)	Na_2_O (%)	K_2_O (%)	SO_3_ (%)
71.40	1.71	0.06	0.06	9.79	3.28	13.17	0.31	0.24

ZnO (99.0% wt. purity) in 5.0, 7.5 and 10.0% amounts was added to the glass batch by reducing the amounts of SiO_2_, CaO, and MgO. The antibacterial glass compositions are listed in Table 2.

**Table 2:** Compositions of Antibacterial Glass Samples Doped with 5.0, 7.5, and 10.0% ZnO (in wt. %)

SiO_2_ (%)	Al_2_O_3_ (%)	Fe_2_O_3_ (%)	TiO_2_ (%)	CaO (%)	MgO (%)	Na_2_O (%)	K_2_O (%)	SO_3_ (%)	ZnO (%)
71.40	1.71	0.06	0.06	7.29	0.78	13.17	0.31	0.24	5.00
68.90	1.71	0.06	0.06	7.29	0.78	13.17	0.31	0.24	7.50
66.40	1.71	0.06	0.06	7.29	0.78	13.17	0.31	0.24	10.00

Hereafter, three glass batches, a mixture prepared by weighing according to a suitable recipe from raw materials and auxiliary substances that gave the oxides in the structure of the glass, of about 120 g each, which were containing approximately 5.0, 7.5, and 10.0% ZnO, were prepared. A platinum crucible (100% purity) was used for melting the glass batches at 1450 °C for 3 h in an air atmosphere in an electric furnace. The melt was poured into water in order to ensure homogeneity. After crushing and pulverizing, the cast glasses were remelted at the same temperature in the furnace and for the same time period to remove the air bubbles from the melt. Finally, the glasses were annealed at 550 °C for 1 h followed by slow cooling to room temperature to remove thermal residual stress.

### 2.2 Characterization of Antibacterial Glasses

Solid medium was used to evaluate the antibacterial properties of glasses against gram-negative bacteria (*Escherichia coli* ATCC 25922). ISO 22196:2011 “Measurement of antibacterial activity on plastics and other non-porous surfaces” was selected as the method for antibacterial testing, to obtain more reliable results. This antibacterial test method was used to test the antibacterial activity of 12 glass samples against “*Escherichia coli*”. The glass samples were initially inoculated with approximately 1 × 10^5^ bacteria/mL of inoculum and coated with a piece of sterile film. After 24 h of incubation at 37 °C, the films were removed from the control and test samples and placed into a phosphate buffered saline solution. Then, *E. coli* counting was performed by serial dilution plating.

The inductively coupled plasma–mass spectrometer (ICP-OES, Perkin Elmer Avio 200, 4% CH_3_COOH concentration, ISO 6486) was used to measure release of ions from the antibacterial glass into acetic acid and water. The tests for antibacterial glasses were performed according to 84/500/European Economic Community (EEC) Directive and British Standards (BS) 6748. In accordance with the 84/500/EEC Directive and BS 6748 standard, ICP-OES was used to determine the release of zinc from the inner surface of the glassware intended to come into contact with foodstuffs in 4% (v/v) acetic acid and water at 22 ± 2 °C and for 24 ± 0.5 h.

The determination and classification of the alkali strength of the glass samples were performed by the application of the ISO 695 “Glass Resistance to attack by a boiling aqueous solution of mixed alkali - method of test and classification” standard. This method consists of determination and classification of the resistance of the glass samples, which interact with sodium hydroxide and sodium carbonate aqueous solution boiling at 102.5 ± 0.5 °C for 3 h according to the mass loss on the unit surface.

X-ray diffraction (XRD) analyzer was used to observe the amorphous structure of the obtained glass (Panalytical Empyrean XRD, 45 mA, 40 kV, scan range: 10–60°, step size: 0.013°).

X-ray photoelectron spectroscopy (XPS) analysis was used to determine the energy levels of oxide, which is important for the antibacterial feature of glasses. For this analysis, Thermo Scientific K-Alpha spectrometer was used. Between the sample surface and the axis of the analyzer lens, there is an aluminum anode (Al Kα = 1468.3 eV) at an electron take-off angle of 90° on the spectrometer. Charging was avoided by using a flood gun. The top surface from any organic impurities was cleaned by using accelerated Ar ions at 3000 eV for 30s. An Avantage 5.9 data system was used to record the spectra.

The reflection (*R*%), transmittance (*T*%), and absorption (*A*) measurements of reference and antibacterial glass were determined by using a PE Lambda 900/950 ultraviolet–visible region-near infrared (UV–vis–NIR) spectrophotometer. This device is a dual-monochromator, double-beamed, and computer-controlled type spectrophotometer, which is used to determine reflection, transmittance, and absorption measurement values in the UV–vis–NIR of the spectrum, 185–3200 nm (nanometer) in the spectral range of 200–2500 nm when using the collector spheres. This device was operated by the UV WinLab software program, which allows the use of four different methods.

Mettler Toledo density kit, which uses water as buoyancy liquid and Archimedes’ principle, was selected to measure the densities of antibacterial and reference (non-antibacterial typical container glass) glasses at room temperature.

Dilatometer (Netzsch DIL 402 PC) was used to detect the thermal expansion coefficients of the antibacterial and reference glasses. The dilatometer can reach a maximum temperature of 1100 °C. The coefficient of thermal expansion in glass samples was measured between 0 and 300 °C.

Nanoindenter was used to measure the hardness and reduced elastic modulus values (M1, NANOVEA). Indentations were performed to a maximum load of 300 mN at loading and unloading rates of 600 mN/mN. The indentation was performed by a Berkovich tip calibrated on fused silica, and 10 indents were performed on each sample.

In the scratch test, a diamond tip with a certain geometry (angle = 120° and radius = 200 μm) was first scanned by selecting the lowest possible load (starting load =1 N and final load =30 N) in order to avoid permanent damage to the surface. A scratch test was initiated according to the predetermined test parameters (upload speed = 15 N/min, scratch length = 10 mm and number of scratches = 5). The critical force value (*L*_C_) at which the first damage (*L*_C1_) started was determined when the load was applied on the sample. At the *L*_C1_ value, the first ring-shaped deformations called Hertzian fractures started; as the load increased, these fractures intensified and turned into severe fractures at *L*_C2_ points, and these values were recorded.

### 2.3 High Temperature Melting Observation

**Figure 2:**
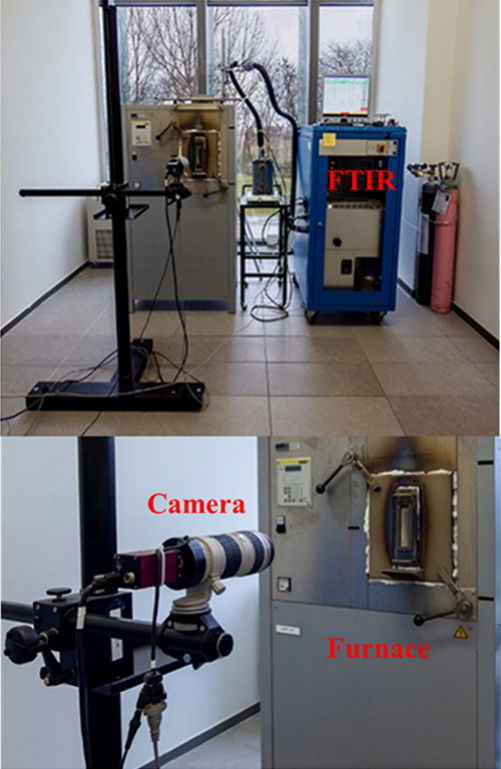
High Temperature Melting Observation System (HTMOS).

Melting properties of the glass composition (5% ZnO) were also examined in comparison with the High Temperature Melting Observation System (HTMOS). The batch, which was prepared before, was melted in a silica tube, and the whole melting process was recorded using a camera in the system, seen in Figure 2. Besides these transactions, Fourier transform infrared (FTIR) spectroscopy was used to measure the quantity of CO_2_ gas, which is important for melting.

## 3 Results and Discussion

### 3.1 Antibacterial Tests of the Obtained Glass Samples

**Figure 3:**
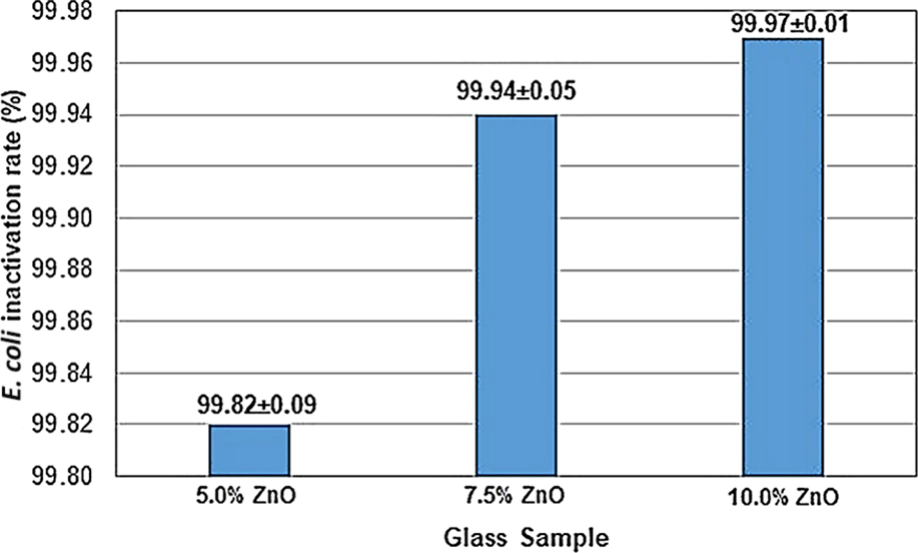
Antibacterial test results for glass samples doped with 5.0, 7.5, and 10% ZnO.

Figure 3 shows the antibacterial test results of the obtained glass samples. As seen in Figure 3, the antibacterial activities of glasses doped with 5.0, 7.5, and 10.0% ZnO were at the desired level. The results indicated that the inactivation rate for*E. coli* of all glasses was higher than 99.82% (>2.8 log). Although the glass sample doped with 10% ZnO having the best antibacterial effect, the glass doped with 5% ZnO was chosen in order to decrease the raw material cost of the glass product. Therefore, characterization tests were applied on only the glass doped with 5% ZnO for the following studies.

Applerot et al. used ultrasonic irradiation to coat the glass with ZnO and give it to the antibacterial feature, but the coating applied to the glass resulted in low antibacterial activity against *E. coli*.^33^ Esteban-Tejeda et al. reported to achieve the desired antibacterial activity against *E. coli*, but the amount of ZnO used in the batch composition was three times more than the amount used in our study.^31^ In another study, there is an antibacterial glass composition doped with the same amount of ZnO. When the antibacterial properties of the glass obtained by using this glass composition were examined, it was seen that a similar antibacterial effect result was obtained. However, there was an additional copper oxide in this batch composition compared to the batch composition in our study.^34^ Therefore, our study provides an important advantage in terms of raw material costs.

### 3.2 Ion Release and Alkali Resistance Test Results of the Antibacterial Glass Sample

Afterwards, the toxic properties of the glass sample doped with 5.0% ZnO were determined with the ICP-OES test. The test was performed twice in the same conditions. One sample was kept in 4% (v/v) acetic acid for 1 day, and the other sample was kept in 4% (v/v) acetic acid for a week at 22 °C (±2 °C). The same experimental conditions were also applied to the samples kept in water. The maximum zinc levels that should be taken in terms of human health on the basis of age and gender are given in Table 3.^35^ According to these values, the toxic values of the obtained glass sample doped with 5.0% ZnO were found to be harmless to human health, as seen in Table 4.

**Table 3:** Tolerable Upper Intake Levels (ULs) for Zinc [^35^]

age	male	female
0–6 months	4 mg	4 mg
7–12 months	5 mg	5 mg
1–3 years	7 mg	7 mg
4–8 years	12 mg	12 mg
9–13 years	23 mg	23 mg
14–18 years	34 mg	34 mg
19+ years	40 mg	40 mg

**Table 4:** ICP-OES Test Results for Glass Samples Doped with 5.0 wt % ZnO

sample definition	released amount of zinc ion (μg/dm^2^)
glass with 5% ZnO pending in acetic acid for 1 day	0.80
glass with 5% ZnO pending in acetic acid for 1 week	1.41
glass with 5% ZnO pending in water for 1 day	0.29
glass with 5% ZnO pending in water for 1 week	0.68

The weight loss of the glass sample containing 5.0% ZnO was determined as 76.07 mg/dm^2^. Therefore, the alkali resistance class of glass was found to be A2 and the alkali resistance characteristic was found to be medium level degradation, according to Alkali Resistance Test Limits, which is suitable to be used as a glass container.

### 3.3 Characterization of the Antibacterial Glass Sample

**Figure 4:**
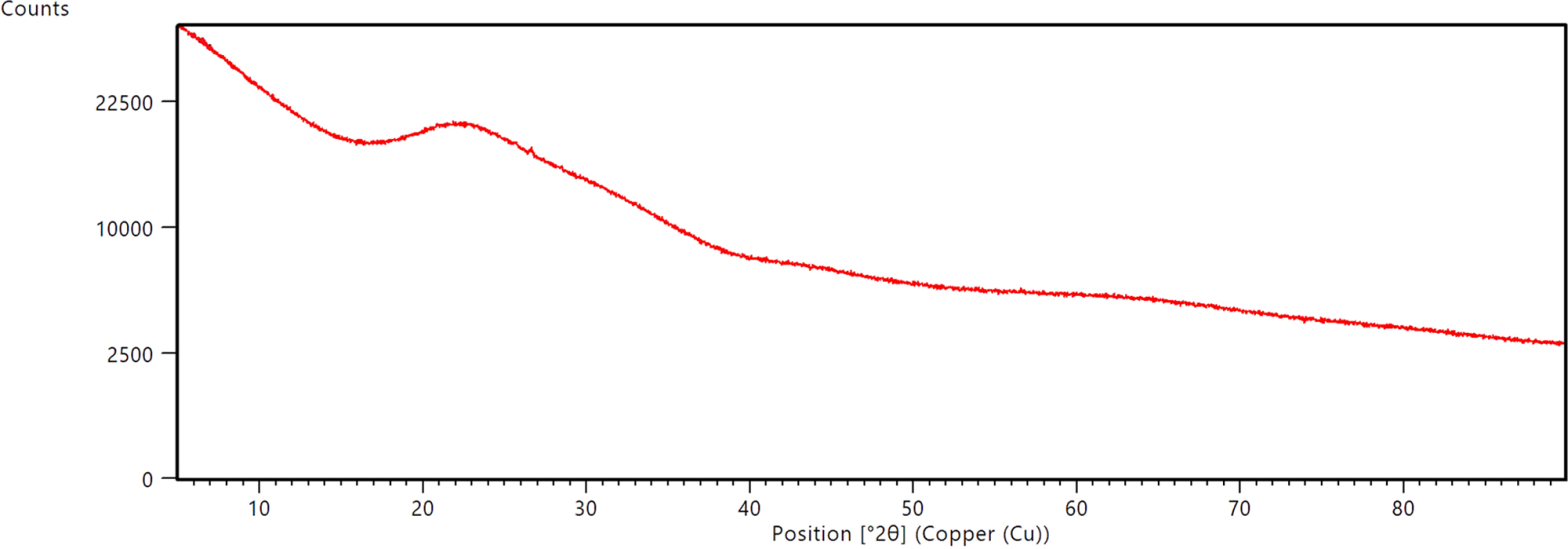
XRD result for the glass sample doped with 5% ZnO.

First, XRD analysis was performed to see the phase structure of the glass. It is seen that there is a characteristic bell curve for the amorphous structure shown in Figure 4. It means that the glass doped with 5.0% ZnO was completely amorphous. The amorphous structure was achieved even in glass structures with a ZnO content of more than 5.0% (in wt. %).^31^ This shows that the 5.0% ZnO (in wt. %) content does not cause any crystallization during the glass production process.

**Figure 5:**
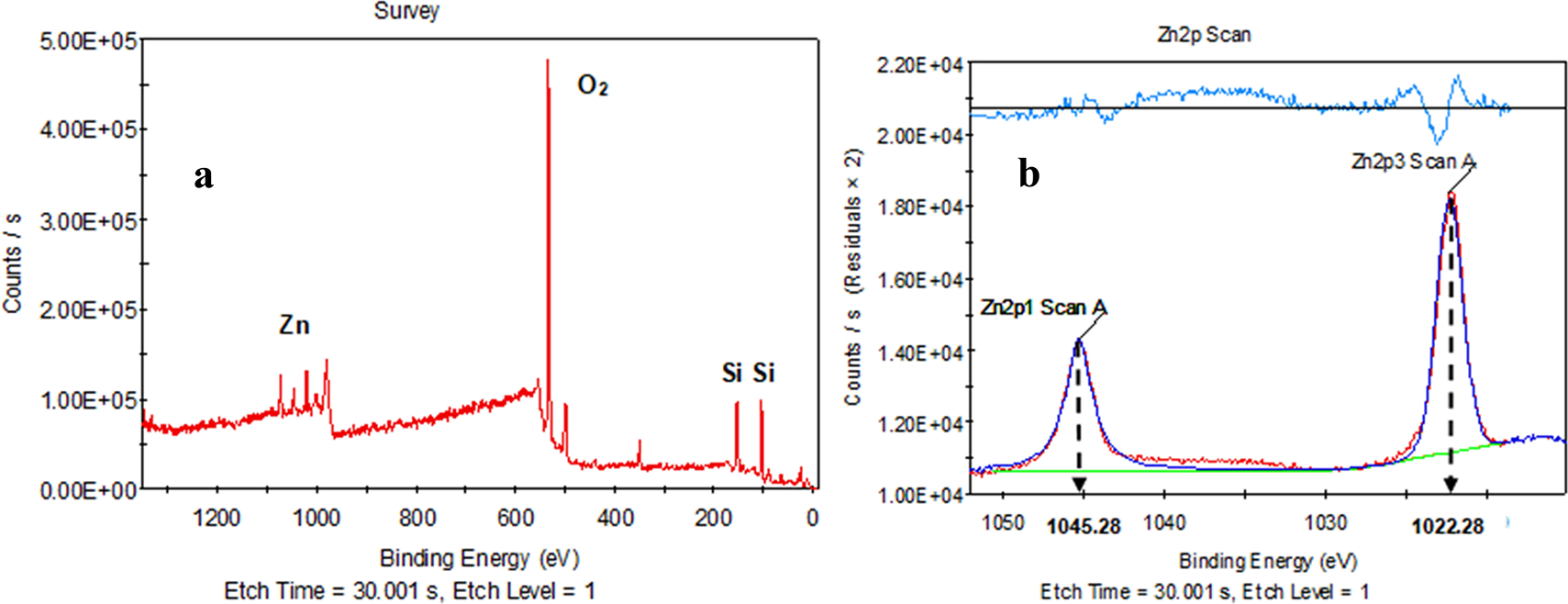
(a) XPS analysis and (b) zinc energy range graph of the glass sample doped with 5% ZnO.

XPS analysis was also applied to the sample in addition to XRD analysis, to determine the energy levels of ZnO. Figure 5a shows that oxygen and silica have the most prominent peaks. ZnO covers the range of 1020–1050 eV, and the expanded graph of this region is shown in Figure 5b. XPS analysis results indicated that ZnO was successfully added into the glass doped with 5% ZnO. This result also confirmed the antibacterial property of the glass sample since ZnO was detected on the surface of the glass sample.

Moreover, additional tests were performed on the antibacterial glass sample to be able to compare the physical, thermal, and optical properties of the reference glass (non-antibacterial typical container glass) with those of the antibacterial glass (5.0% ZnO). Table 5 shows the obtained results. The physical, thermal, and optical properties of antibacterial glass and the reference glass (non-antibacterial, soda lime glass) were found to be close to each other. Furthermore, the antibacterial glass was found to be colorless, which is an important property for a glass container.

**Table 5:** Physical and Optical Test Results for the Reference and Antibacterial (5.0 wt % ZnO) Glass Samples

	reference	antibacterial
thermal expansion coefficient (10^–7^/°C)	86.5	86.1
density (g/cm^3^)	2.493	2.523
refractive index	1.5200	1.5191
color parameters (std. 3 mm)		
brightness (%)	72.0	90.5
dominant wavelength (nm)	556.5	577.2

Sayyed et al. performed a study using ZnO in soda lime glass. The results of the study showed that the densities of the glass without ZnO and glass doped with 10% ZnO were measured as 2.520 and 2.570 g/cm^3^, respectively.^36^ Although the zinc amount of the glass doped with 5% ZnO was between these two values, the density of the glass was very close to density of soda lime glass. Other studies also reported that the glass samples appeared transparent like the other antibacterial zinc glasses.^37^

The preliminary melting behavior was also observed by measuring the viscosity of the glasses. The viscosity values were found to be slightly different, as seen in Table 6. However, this difference is seen to decrease when the softening temperature is reached.

**Table 6:** Viscosity Measurement Results for the Reference and Antibacterial (5.0 wt % ZnO) Glass Samples

	temperature (°C)	
viscosity	reference	antibacterial
log η = 2.25 (±0.018) (melting temp.)	1372	1435
log η = 2.50 (±0.014)	1309	1367
log η = 2.75 (±0.009)	1252	1306
log η = 3.00 (±0.011) (gob temp.)	1200	1250
log η = 3.25 (±0.012)	1154	1200
log η = 3.50 (±0.015)	1111	1153
log η = 4.00 (±0.016)	1036	1071
log η = 7.65 (softening temp.)	734 (±2.3)	738 (±2.3)
working range (WR) *T*_logη = 3_ – *T*_logη = 7.65_	1372	1435

**Figure 6:**
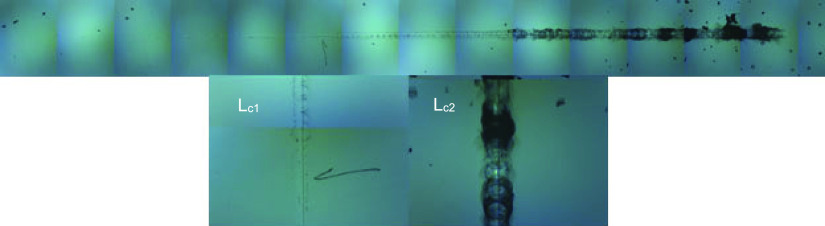
Surface morphology of the glass doped with 5.0% ZnO.

Lastly, the mechanical behavior was determined with hardness and elastic modulus of the glasses. The hardness and elastic modulus of the antibacterial glass were the same as the hardness and elastic modulus of the reference (soda lime) glass. Also, the scratched image of the glass doped with 5.0% ZnO is given in Figure 6. The first deformations of the glass were seen in the *L*_c1_ part of the figure. In the *L*_c2_ part of the figure, the severe breakage that occurred in the glass with the increase of the load is seen. However, it was determined that the antibacterial glass was scratched at lower loads compared to the reference glass (Table 7). Overall results indicated that the physical, optical, thermal, and mechanical properties of the antibacterial glass sample and commercial soda lime glass are the same except scratch resistance.

**Table 7:** Mechanical Test Results for the Reference and Antibacterial (5.0 wt % ZnO) Glass Samples

	reference	antibacterial
hardness (kg/mm^2^)	596 ± 29.84	596 ± 29.84
elastic modulus (GPa)	5.84 ± 0.9	5.84 ± 0.9
scratch test		
*L*_C1_ (N)	13.20 ± 3.67	3.90 ± 0.44
*L*_C2_ (N)	23.88 ± 2.37	19.25 ± 1.27

### 3.4 High Temperature Melting Observation System

**Figure 7:**
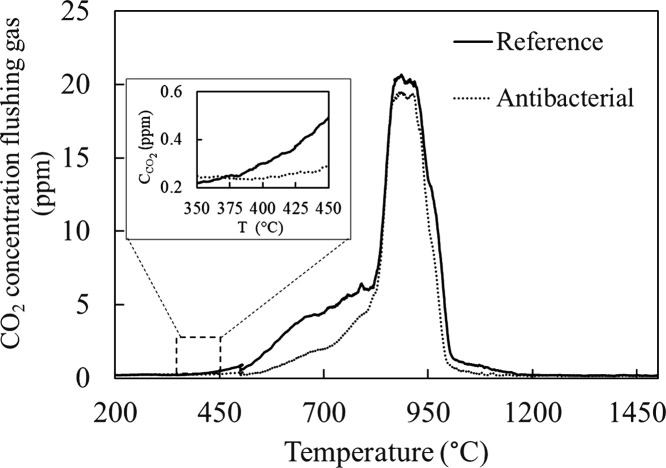
CO_2_ release during HTMOS measurements for the reference and antibacterial (doped with 5% ZnO) compositions.

**Figure 8:**
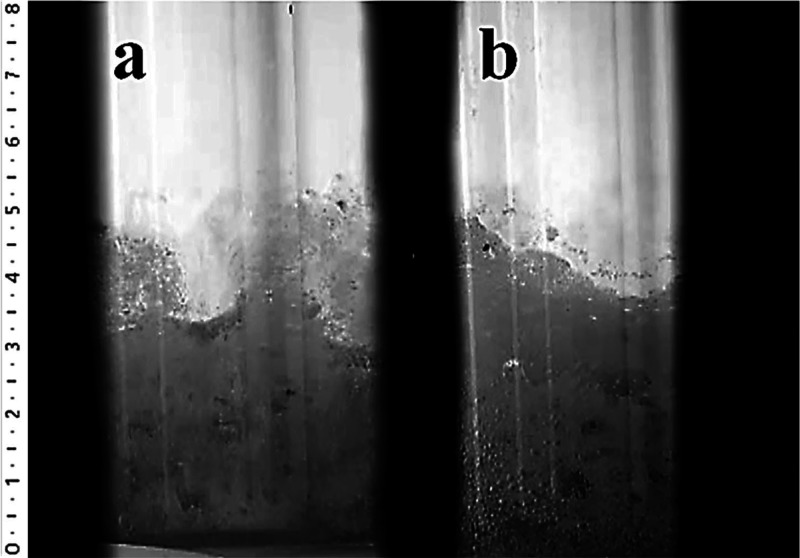
Fining states of the (a) reference and (b) antibacterial (doped with 5% ZnO) compositions at 1250 °C.

**Figure 9:**
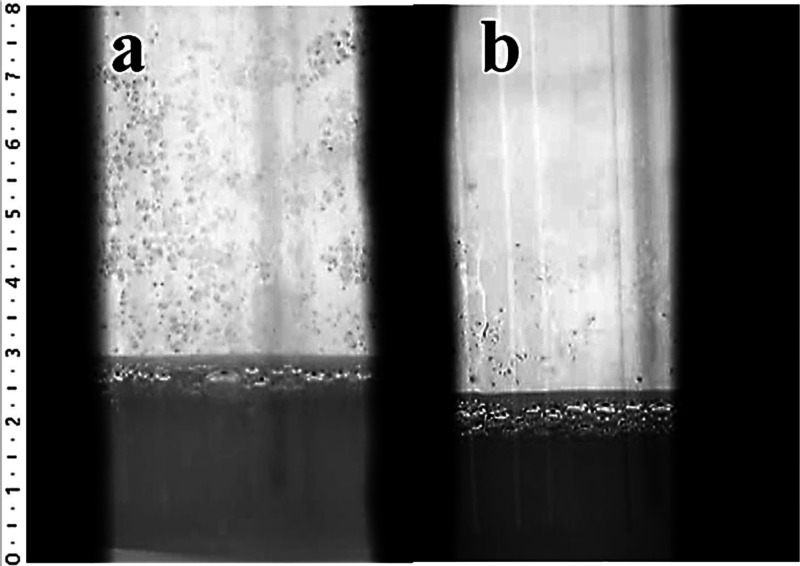
Fining states of the (a) reference and (b) antibacterial (doped with 5% ZnO) compositions at the end of the experiment.

It is the first time that HTMOS was performed on the ZnO-doped antibacterial soda lime glass obtained by the conventional melting method. The CO_2_ results in Figure 7 showed that the melting reactions of the reference composition started before the antibacterial composition was doped with 5.0% ZnO. Also, according to Supporting Videos 1 and 2, the fining of the antibacterial composition doped with 5.0% ZnO begins faster than the reference glass (shown in Figure 8), but it is shown that the fining of the reference glass appears to be better than the antibacterial glass from the surface of both glasses at the end of the experiment (Figure 9). The explanation for this is the low viscosity of the glass. When the viscosity of the glass is low, it is easier for the gases to come up to the surface of glass and to be thrown out.

Arslan et al. and Demirok et al. used HTMOS to perform detailed melting behavior experiments of various soda lime glass compositions. The results showed that antibacterial soda lime glass obtained by the conventional melting method has a similar melting behavior with these compositions.^38,39^ This result shows that there is no need to change the production parameters and furnace design for the production of antibacterial glass in soda lime glass furnaces.

### 3.5 Cost Analysis

Antibacterial glasses are frequently preferred especially in health, household goods, and packaging. The income to be obtained from health practices all over the world is expected to increase above 7% by 2023. The oncology, burn, and hematology units in the health sector are giving great importance to hygiene, which is the reason to expect that the increase in antibacterial applications in these units will affect the antibacterial glass market positively. In addition, the need for long-term storage of food and beverages increases the importance of antibacterial glasses in the food industry. The antibacterial glass industry will also grow with the desire to increase food quality in restaurants and canteens. In Europe, it is expected that the standards of EN 1650, EN 13697, and EN 1276, which will enable the bactericidal evaluation of foods in the food industry, will enable the development of the antibacterial glass market, and the size of the market will reach 150 million dollars in 2023.^40^

According to energy cost calculated by using experimental viscosity results, the melting temperature of the reference and antibacterial compositions are very close to each other. From this result, it can be said that the energy costs of two compositions are nearly the same. However, because of the price of ZnO, the raw material cost of the antibacterial composition is higher than that of the raw material cost of the reference composition (soda lime glass for glass containers). When raw material and energy costs are evaluated together (due to the Şişecam privacy policy, data cannot be given here), the cost is a bit high because the batch composition of the antibacterial glass contains precious metal. However, soda lime glass is compared with a glass having antibacterial properties in this cost analysis. Considering the markets of soda lime glass containers and the antibacterial glass materials, it is clear that the product sales prices will be very different. Therefore, antibacterial soda lime glass materials can be bearable in the future as it will bring a significant advantage. Considering all test results and cost analysis of the colorless antibacterial glass sample, it is concluded that the obtained antibacterial glass can be used in the food industry as a glass container and in the health sector such as architectural glass of hospitals and pharmaceutical glass packaging and antibacterial glassware products at our homes.

## 4 Conclusions

In this study, glass with an antibacterial property (99.82% activity to *E. coli*) derived from soda lime glass composition was obtained by adding ZnO to the batch. ICP-OES results showed that release values (max: 1.41 μg/dm^2^) were lower than toxic values (min: 4 mg). Additionally, the degradation rate of antibacterial glass was shown to be lower against the alkali solution so it was defined as class A2. The physical (density: 2.523 g/cm^3^), thermal (thermal expansion coefficient: 86.1 × 10^–7^/°C), and optical properties (dominant wavelength: 577.2) of the antibacterial glass and reference glass were nearly the same. The antibacterial glass was found to be more viscous than the reference (non-antibacterial) glass. An HTMOS study indicated that the antibacterial composition can be produced in the glass industry and there is no need to change the furnace design and production parameters. Although the production cost of antibacterial glass is a bit higher than that of the reference composition, the use of antibacterial glass can be considered in vital areas for humans such as the healthcare sector and food industry and in daily lives. Considering the markets of antibacterial glass containers, the obtained antibacterial soda lime glass’ price can be bearable with the products used in the market. It is known that the antibacterial soda lime glass materials were generally produced by using ion exchange. However, there are some important constraints with the production of these materials such as changing glass production processes or adapting new systems to the process and short-term antibacterial effect. This study brings a new perspective in the soda lime glass industry by adding long-lasting antibacterial properties in soda lime glass with the classical melting method without changing the whole process. This is a novelty in this field without any extra cost and without the need to change the production parameters and furnace design for the production of antibacterial glass in soda lime glass furnaces.

## Supporting Information Available

Video 1: Melting and fining reactions of reference soda lime glass (MP4)
Video 2: Melting and fining reactions of antibacterial soda lime glass (MP4)
